# Supplementary material for: Effect of Snowmelt on Groundwater Bacterial Community Composition and Potential Role of Surface Environments as Microbial Seed Bank in Two Distinct Aquifers from the Region of Quebec, Canada
Source: Microorganisms. 2023 Jun 8;11(6):1526. doi: 10.3390/microorganisms11061526 (PMC10304690; doi:10.3390/microorganisms11061526)
Supplement: Supplementary file 1 [file microorganisms-11-01526-s001.zip › microorganisms-2345813-supplementary.pdf]

microorganisms

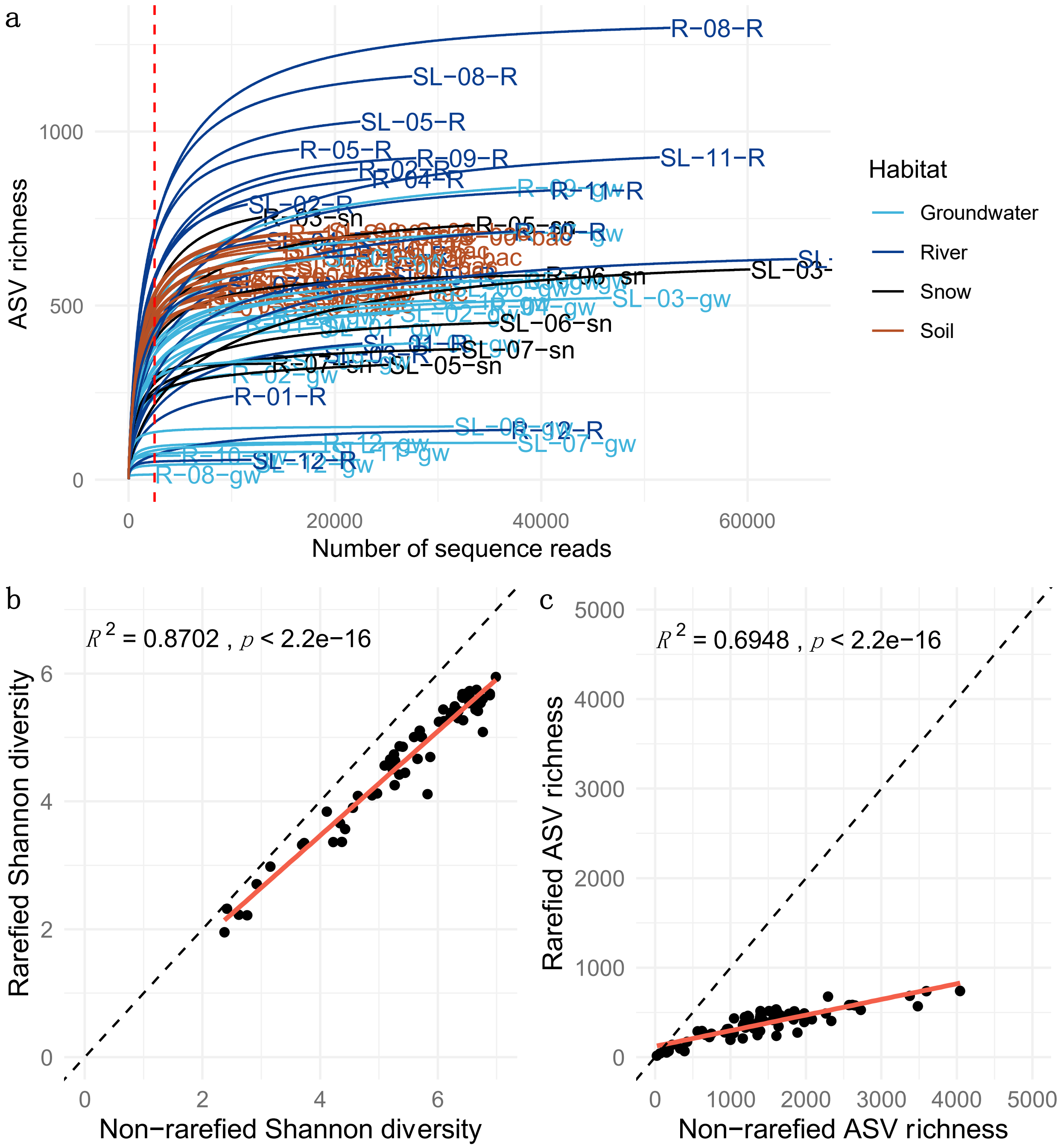

Figure S1. Rarefaction curve and Spearman correlation between diversity and richness of rarefied and non-rarefied samples. (**a**) Rarefaction curve displaying the number of ASVs against the number of sequences for each sample. The vertical red dashed line indicates the rarefaction depth used for all samples (i.e., 2,530 reads per sample). (**b**) Correlation between calculated Shannon diversity of rarefied and non-rarefied samples. (**c**) Correlation between ASV richness of rarefied and non-rarefied samples. Linear regression is showed in red, and the dotted line represents a one-to-one theoretical relationship where rarefaction has no impact on diversity and richness. The R^2 denotes the Spearman correlation coefficient (n=71).

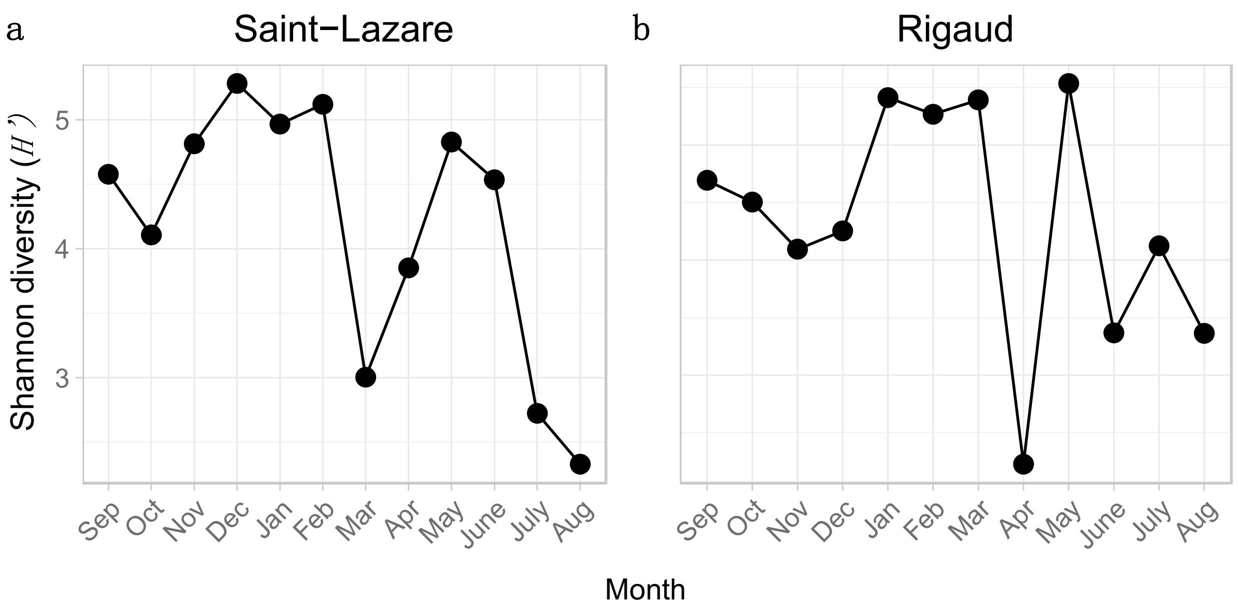

Figure S2. Shannon diversity of groundwater bacterial communities of both aquifer across the sampling period. (**a**) The Saint-Lazare aquifer. (**b**) The Rigaud aquifer. Only one sample was collected for each month.

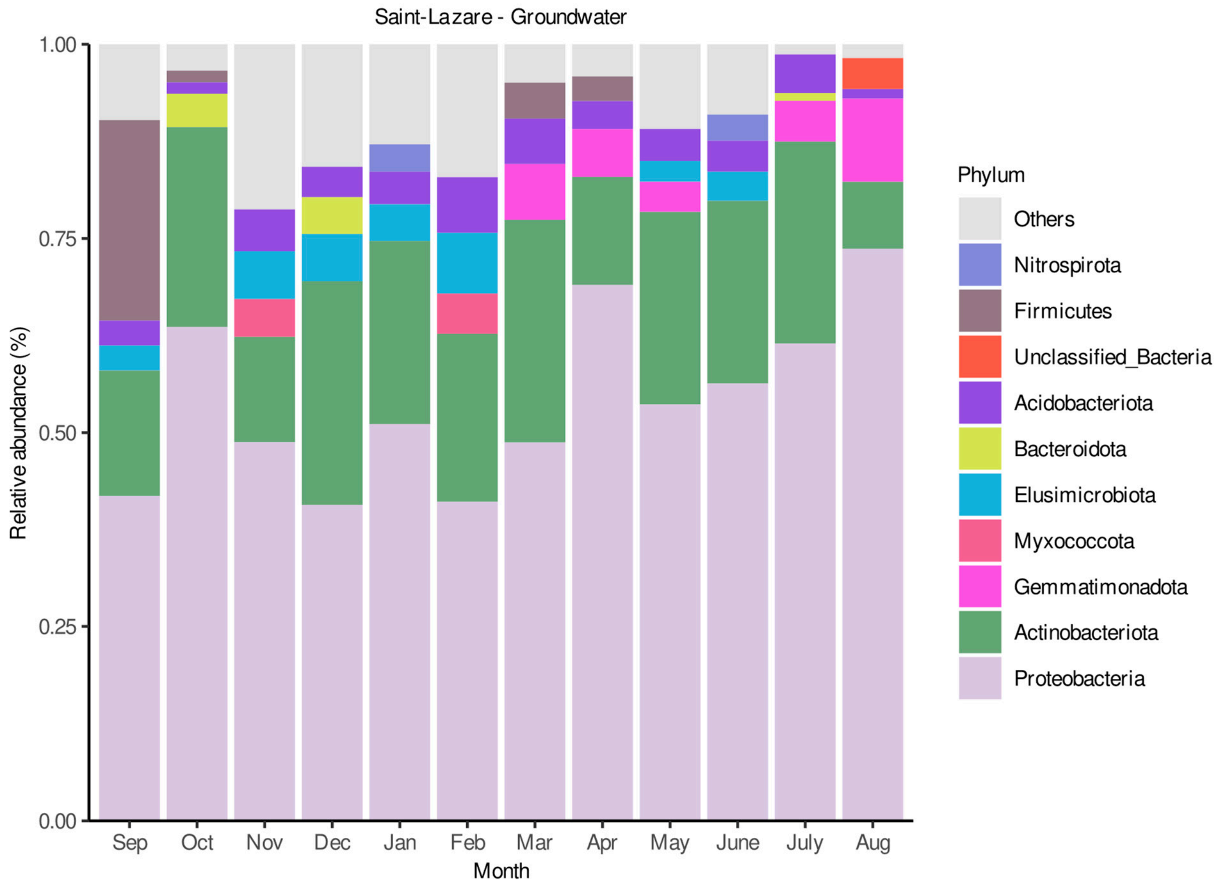

Figure S3. Relative abundance (%) of amplicon sequence variant (ASV) at the phylum level in groundwater samples collected monthly at the Saint-Lazare site. For each month, only the five most abundant families are colored.

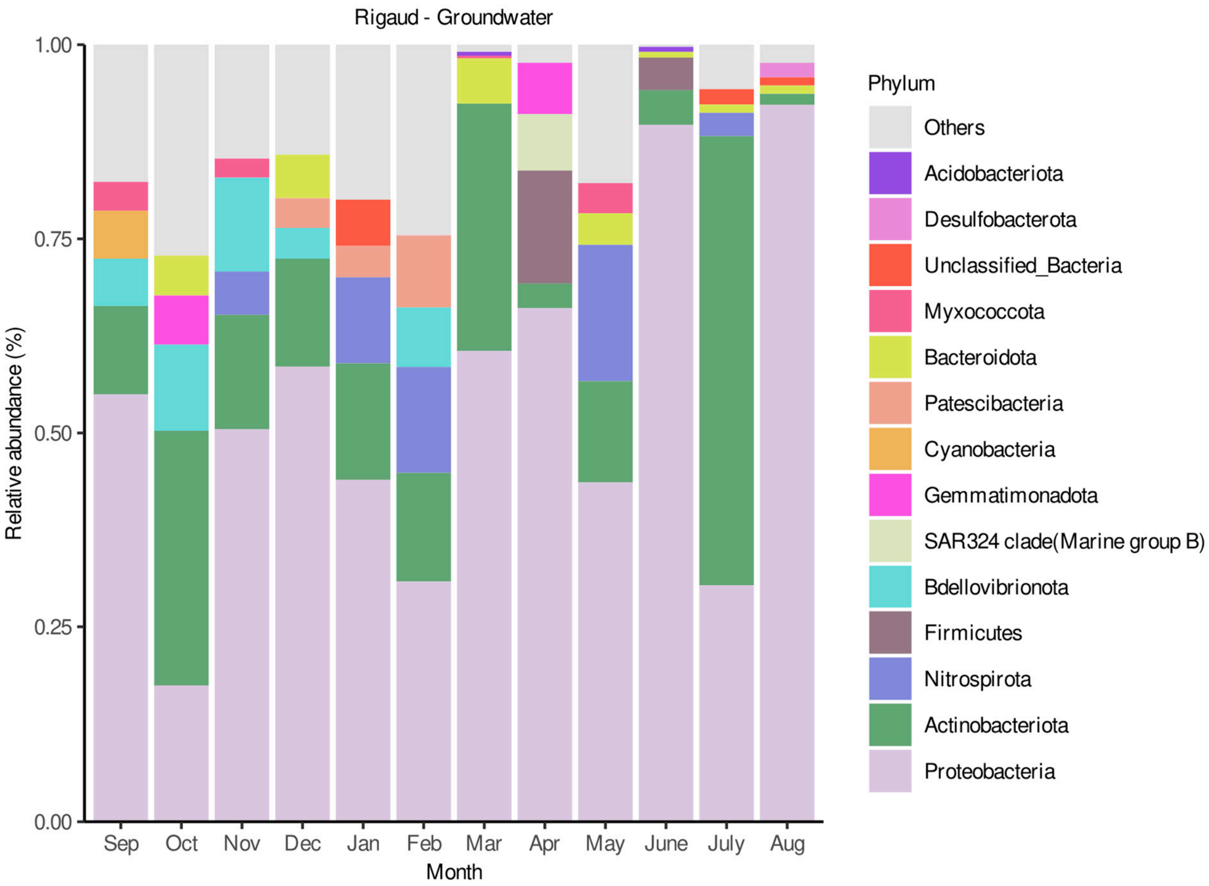

Figure S4. Relative abundance (%) of amplicon sequence variant (ASV) at the phylum level in groundwater samples collected monthly at the Rigaud site. For each month, only the five most abundant families are colored.

Table S1. Maximum and minimum daily average of each month for groundwater level and temperature from both aquifers. Data was collected by a probe installer semi-permanently in each well. Hourly measures were averaged for each date. Level indicates the height in meter (m) of the water above the probe.

	Saint-Lazare				Rigaud			
	Groundwater temperature (°C)		Groundwater level (m)		Groundwater temperature (°C)		Groundwater level (m)	
**Month**	**Max**	**Min**	**Max**	**Min**	**Max**	**Min**	**Max**	**Min**
**Sep.**	7.02	6.87	3.20	3.06	10.19	9.15	0.54	0.40
**Oct.**	7.19	7.02	3.06	2.95	10.78	10.22	0.75	0.38
**Nov.**	7.34	7.20	3.13	2.94	10.87	10.31	1.12	0.94
**Dec.**	7.40	7.34	3.12	3.03	10.27	8.84	1.02	0.90
**Jan.**	7.41	7.39	3.08	3.01	7.52	6.75	1.84	1.42
**Feb.**	7.39	7.31	3.05	2.98	6.76	6.69	1.41	1.13
**Mar.**	7.31	5.59	3.40	2.74	6.69	4.32	2.56	1.12
**Apr.**	5.65	5.22	3.76	3.07	4.27	3.61	2.56	1.83
**May**	6.05	5.57	3.78	3.68	4.67	3.71	1.81	1.27
**June**	6.29	6.07	3.68	3.54	6.36	4.71	1.25	0.90
**July**	6.41	6.30	3.55	3.40	8.22	6.42	0.89	0.66
**Aug.**	6.54	6.41	3.39	3.24	9.87	8.28	0.69	0.60

Table S2. Physicochemical qualities of groundwater. Dissolved inorganic carbon (DIC); Dissolved organic carbon (DOC); Nitrites (NO_2); Nitrates (NO_3); Ammonium and ammonia (NH_x); Dissolved oxygen (DO).

	Saint-Lazare						Rigaud					
**Month**	**DIC (mg/L)**	**DOC (mg/L)**	**NO_2 (mg/L)**	**NO_3 (mg/L)**	**NH_x (mg/L)**	**DO (%)**	**DIC (mg/L)**	**DOC (mg/L)**	**NO_2 (mg/L)**	**NO_3 (mg/L)**	**NH_x (mg/L)**	**DO (%)**
**Sep.**	2.10	0.50	0.00	0.04	0.01	101.20	60.90	1.10	0.00	1.16	0.02	3.80
**Oct.**	2.30	0.50	0.00	0.05	0.01	100.50	67.20	1.00	0.00	5.52	0.02	11.00
**Nov.**	2.00	0.30	0.00	0.05	0.00	89.90	67.20	1.10	0.00	9.35	0.03	11.00
**Dec.**	2.52	0.57	0.00	0.05	0.00	101.70	67.17	1.21	0.00	7.94	0.01	19.60
**Jan.**	1.90	0.50	0.00	0.05	0.00	103.50	65.10	1.20	0.00	5.85	0.00	3.12
**Feb.**	2.20	0.00	0.00	0.05	0.01	100.00	68.20	1.80	0.00	4.18	0.00	3.70
**Mar.**	5.00	0.60	0.00	0.05	0.00	99.30	107.50	1.20	0.04	6.69	0.01	30.50
**Apr.**	2.60	0.00	0.00	0.08	0.01	101.00	58.50	0.20	0.00	4.74	0.02	45.70
**May**	2.60	0.00	0.00	0.07	0.07	100.70	65.40	0.50	0.00	6.19	0.01	18.50
**June**	2.80	0.00	0.00	0.09	0.01	98.80	68.10	0.00	0.01	6.56	0.01	18.80
**July**	2.20	0.40	0.00	0.05	0.01	99.10	68.30	0.90	0.01	2.64	0.00	36.70
**Aug.**	2.10	0.20	0.01	0.07	0.01	94.30	60.40	0.60	0.01	1.18	0.00	9.70

Table S3. PERMANOVA results for all habitats. Variation in bacterial community composition explained by habitat type (groundwater, river, soil, snow, discharge) for both sites, tested by PERMANOVA of Hellinger-transformed ASV abundance data using the Bray-Curtis dissimilarity index.

		**Df**	**R^2**	**F**	**p**
**Saint-Lazare**	Habitat	6	0.5129	7.0189	0.001
	Residual	40	0.48713		
	Total	46	1		
**Rigaud**	Habitat	3	0.4283	7.7403	0.001
	Residual	31	0.5717		
	Total	34	1		

Table S4. PERMANOVA results for groundwater. Variation in bacterial community composition in aquifer explained by period (pre-recharge, post-recharge) for both sites, tested by PERMANOVA of Hellinger-transformed ASV abundance data using the Bray-Curtis dissimilarity index.

		**Df**	**R^2**	**F**	**p**
**Saint-Lazare**	Period	1	0.2535	3.3965	0.004
	Residual	10	0.7464		
	Total	11	1		
**Rigaud**	Period	1	0.1706	2.0571	0.033
	Residual	10	0.8293		
	Total	11	1		

Table S5. Shannon diversity of groundwater bacterial communities of both aquifer across the sampling period.

**Month**	**Saint-Lazare**	**Rigaud**
September	4.57791	4.69314
October	4.10644	4.50448
November	4.81330	4.09395
December	5.28176	4.25329
January	4.96625	5.41253
February	5.11970	5.26758
March	3.00358	5.39254

April	3.85182	2.22596
May	4.82736	5.53529
June	4.53546	3.36711
July	2.72338	4.12376
August	2.32898	3.36365

Table S6. Average contribution (%) of each source to groundwater bacterial diversity of both aquifers. Potential sources for both aquifers include groundwater, snow, and soil. For the Rigaud aquifer river were also considered given the groundwater-river connections. For each source, only samples collected up to three months prior to groundwater sample assigned as sink were included.

Aquifer	Sink	Groundwater	River	Snow	Soil	Unknown
**Saint-Lazare**	Oct. groundwater	68.77	NA	NA	4.33	26.90
	Nov. groundwater	68.50	NA	NA	2.26	29.24
	Oct. groundwater	68.77	NA	NA	4.33	26.90
	Dec. groundwater	52.50	NA	1.85	9.73	35.91
	Jan. groundwater	81.09	NA	0.63	1.15	17.13
	Feb. groundwater	79.51	NA	0.30	1.17	19.01
	Mar. Groundwater	84.98	NA	0.34	0	14.68
	Apr. groundwater	61.96	NA	18.93	NA	19.11
	May groundwater	61.21	NA	1.00	1.94	35.85
	July groundwater	87.07	NA	NA	0.06	12.88
	June groundwater	63.38	NA	0	2.55	34.07
	Aug. groundwater	80.72	NA	NA	0	19.28
	**Average**	71.54	NA	3.29	2.50	24.25
	**Standard deviation**	10.92	NA	6.92	2.85	8.29
**Rigaud**	Oct. groundwater	17.10	0.25	NA	0.43	82.23
	Nov. groundwater	27.79	0.64	NA	0.26	71.30
	Dec. groundwater	24.67	0.20	0.04	0.10	74.99
	Jan. groundwater	46.98	1.69	0.89	0.57	49.88
	Feb. groundwater	55.65	1.49	0.16	0.08	42.62
	Mar. groundwater	2.66	5.36	62.85	0.23	28.90
	Apr. groundwater	30.96	0.09	0.18	NA	68.77
	May groundwater	40.72	7.03	1.14	0.33	50.77
	June groundwater	2.59	0	3.29	0	94.12
	July groundwater	62.90	2.59	NA	10.35	24.17
	Aug. groundwater	29.96	0.69	NA	0.00	69.34
	**Average**	31.09	1.82	9.79	1.23	59.73
	**Standard deviation**	19.57	2.34	23.42	3.21	22.17

Table S7. Average contribution (%) of each source to river bacterial diversity of both sites. Potential sources for both aquifers include groundwater, snow, and river. For each source, only samples collected up to three months prior to river sample assigned as sink were included.

Site	Sink	Groundwater	River	Snow	Unknown
**Saint-Lazare**	Oct. river	4.65	18.00	NA	77.35
	Nov. river	4.10	16.75	NA	79.15
	Dec. river	4.37	37.61	0.08	57.94
	Jan. river	4.86	64.27	0.35	30.52
	Feb. river	NA	NA	NA	NA
	Mar. river	3.41	44.88	2.89	48.83
	Apr. river	3.03	53.26	1.36	42.35
	May. river	0.45	14.81	0.08	84.66

	June. river	0.51	24.80	0.00	74.69
	July. river	3.76	26.71	NA	69.53
	Aug. river	0.95	36.22	NA	62.83
	**Average**	3.01	33.73	0.79	62.78
	**Standard deviation**	1.73	16.61	1.14	17.71
**Rigaud**	Nov. river	2.22	21.60	NA	76.18
	Oct. river	15.33	16.36	NA	68.31
	Dec. river	0.97	46.80	2.42	49.80
	Jan. river	0.65	47.59	10.05	41.71
	Feb. river	NA	NA	NA	NA
	Mar. river	NA	NA	NA	NA
	Apr. river	3.78	52.66	2.92	40.64
	May. river	1.71	22.40	0.62	75.27
	June. river	1.12	19.88	0.10	78.91
	July. river	0.93	31.02	NA	68.05
	Aug. river	0.00	64.59	NA	35.41
	**Average**	2.84	33.95	2.88	55.20
	**Standard deviation**	4.51	17.46	3.68	20.98
